# A pelvis MR transformer-based deep learning model for predicting lung metastases risk in patients with rectal cancer

**DOI:** 10.3389/fonc.2025.1496820

**Published:** 2025-02-06

**Authors:** Yin Li, Shuang Li, Ruolin Xiao, Xi Li, Yongju Yi, Liangyou Zhang, You Zhou, Yun Wan, Chenhua Wei, Liming Zhong, Wei Yang, Lin Yao

**Affiliations:** ^1^ Department of Information, The Sixth Affiliated Hospital, Sun Yat-sen University, Guangzhou, China; ^2^ Biomedical Innovation Center, The Sixth Affiliated Hospital, Sun Yat-sen University, Guangzhou, China; ^3^ Department of Information, The Sixth Affiliated Hospital, Sun Yat-sen University Yuexi Hospital, Maoming, China; ^4^ Department of General Practice, The Sixth Affiliated Hospital, Sun Yat-sen University, Guangzhou, China; ^5^ School of Biomedical Engineering, Southern Medical University, Guangzhou, China; ^6^ Guangdong Provincial Key Laboratory of Medical Image Processing, Guangzhou, China; ^7^ Department of Radiology, The Sixth Affiliated Hospital, Sun Yat-sen University Yuexi Hospital, Maoming, China

**Keywords:** rectal cancer, lung metastases, MRI, transformer, deep learning

## Abstract

**Objective:**

Accurate preoperative evaluation of rectal cancer lung metastases (RCLM) is critical for implementing precise medicine. While artificial intelligence (AI) methods have been successful in detecting liver and lymph node metastases using magnetic resonance (MR) images, research on lung metastases is still limited. Utilizing MR images to classify RCLM could potentially reduce ionizing radiation exposure and the costs associated with chest CT in patients without metastases. This study aims to develop and validate a transformer-based deep learning (DL) model based on pelvic MR images, integrated with clinical features, to predict RCLM.

**Methods:**

A total of 819 patients with histologically confirmed rectal cancer who underwent preoperative pelvis MRI and carcinoembryonic antigen (CEA) tests were enrolled. Six state-of-the-art DL methods (Resnet18, EfficientNetb0, MobileNet, ShuffleNet, DenseNet, and our transformer-based model) were trained and tested on T2WI and DWI to predict RCLM. The predictive performance was assessed using the receiver operating characteristic (ROC) curve.

**Results:**

Our transformer-based DL model achieved impressive results in the independent test set, with an AUC of 83.74% (95% CI, 72.60%-92.83%), a sensitivity of 80.00%, a specificity of 78.79%, and an accuracy of 79.01%. Specifically, for stage T4 and N2 rectal cancer cases, the model achieved AUCs of 96.67% (95% CI, 87.14%-100%, 93.33% sensitivity, 89.04% specificity, 94.74% accuracy), and 96.83% (95% CI, 88.67%-100%, 100% sensitivity, 83.33% specificity, 88.00% accuracy) respectively, in predicting RCLM. Our DL model showed a better predictive performance than other state-of-the-art DL methods.

**Conclusion:**

The superior performance demonstrates the potential of our work for predicting RCLM, suggesting its potential assistance in personalized treatment and follow-up plans.

## Introduction

Colorectal cancer ranks third globally in terms of incidence among the most prevalent cancers, with rectal cancer (RC) accounting for approximates 30% of all cases within the colorectal cancer category (1, 2). Due to the unique venous drainage through the iliac vessels in the rectum, RC patients have a significantly higher incidence of lung metastasis than those with colon cancers (3–5). Surgical resection of lung metastasis is an optimal treatment method for RC patients to survive long-term (6), which increases the 5-year survival rate to approximately 50% (7, 8). Nevertheless, the prognosis of RC with metastasis remains poor without timely treatment (9). Thus, timely assessment of lung metastasis in patients with RC (RCLM) is important, which further influences the clinical personalized treatment and follow-up plans.

However, the evaluation of RCLM through long-term follow-up with chest computed tomography (CT) scans may present challenges. Radiologists may encounter difficulties in detecting early metastatic lesions due to their small size and the presence of various benign lesions, which will bring additional ionizing radiation exposure and the costs associated with chest CT in patients without lung metastases and may delay the treatment period (10–12). Therefore, a new diagnostic method is needed for reducing radiation exposure and mitigating treatment delays in patients without lung metastases.

Pelvis magnetic resonance imaging (MRI), which has no radiation exposure, is a standard procedure for the detection and staging of RC (13, 14). Previous studies have highlighted the promising role of T2-weighted image (T2WI) in detecting distant metastasis (DM) in RC patients (15, 16). Besides, diffusion-weighted image (DWI), using differences in water molecules to generate image contrast, has shown improved accuracy in detecting RC patients with DM (17, 18). Artificial intelligence (AI) has shown great success in the detection of liver or lymph node metastasis (19–22). However, few have attempted to evaluate of AI models for predicting RCLM based on pelvis MR images of primary tumor. Although these MRI-based AI methods can potentially predict lung metastasis risk, the inherent locality and the consecutive down-sampling operations in the convolutional neural networks limit the extraction of global spatial dependencies. Moreover, the performance of predicting RCLM based solely on pelvis MRI scans is modest.

In this study, we introduce a pelvis MR transformer-based deep learning (DL) model for predicting RCLM based on T2WI and DWI. Our DL model leverages pre-trained UniMiSS (23), which built upon the ViT framework and trained on medical images, as our primary feature extraction network. Numerous studies have shown that carcinoembryonic antigen (CEA) is a critical biomarker for monitoring recurrence and metastasis in RC patients (14, 32). Therefore, we incorporate clinical information such as CEA, age, and gender due to their stronger association with RCLM, in our DL model to improve the performance of predicting RCLM.

## Materials and methods

### Ethics statement

This single-center retrospective study received approval from our institutional review board and complied with ethical regulations. The requirement for informed consent was waived for this retrospective study of anonymized data.

### Patients

**Figure 1:**
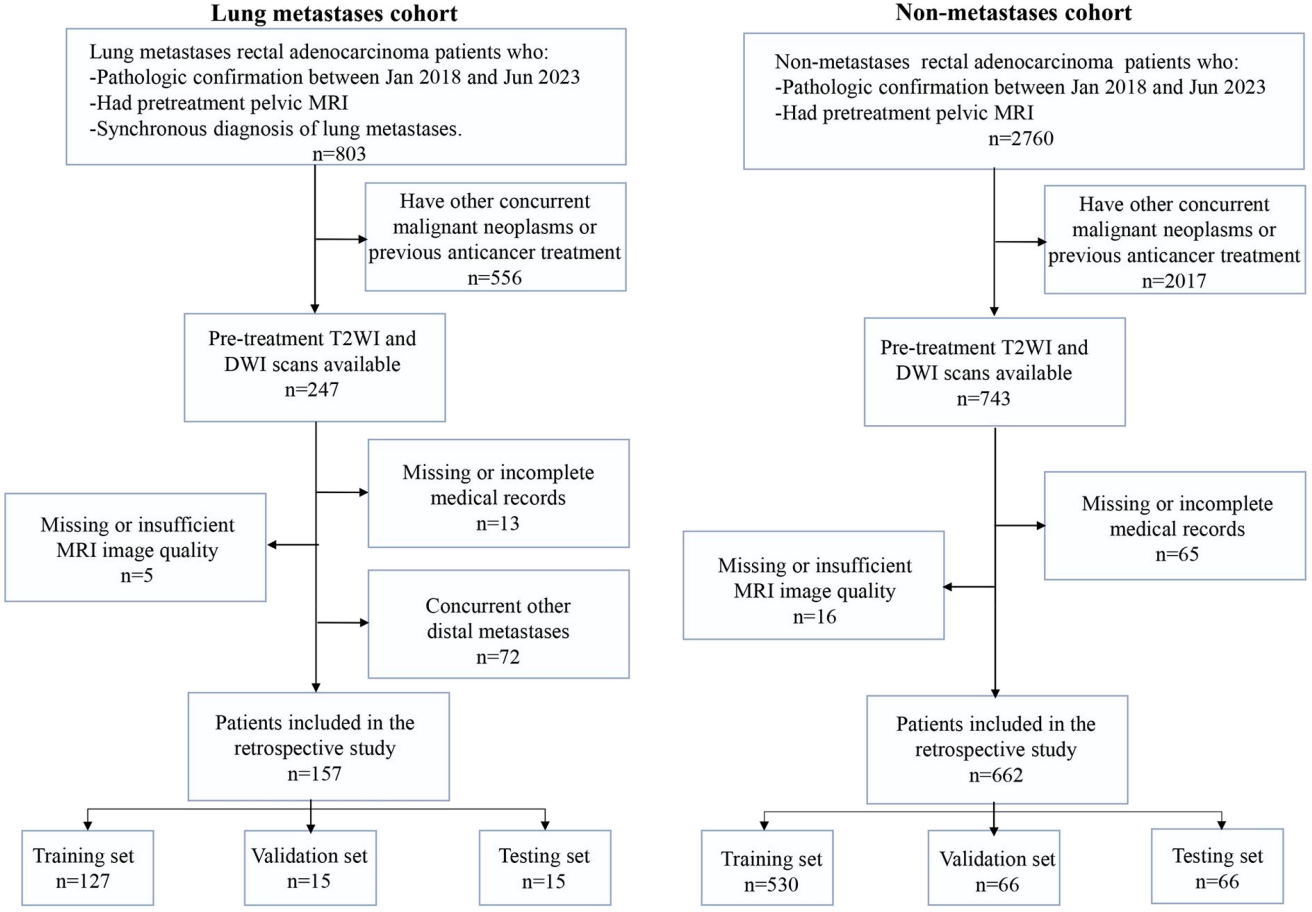
The flowcharts of patient selection.

With the approval of institution ethics committee, we collected two independent patient cohorts. The detailed inclusion and exclusion criteria are shown in **Figure 1**. The lung metastasis cohort of 157 RC patients with lung metastasis risk diagnosed between Jan 2018 and Jun 2023. Inclusion criteria were as follows: (a) pathological confirmation of rectal adenocarcinoma; (b) availability of pre-treatment pelvis MRI scans before the initiation of therapy; and (c) utilization of high-resolution contrast enhanced chest CT scans for lung metastasis diagnosis. The exclusion criteria included: (a) concurrent others primary malignant neoplasms or previous anticancer treatment; (b) missing MRI data or insufficient image quality; (c) missing or incomplete electronic medical records; and (d) simultaneous occurrence of other DM.

Besides, we collected a non-overlapping cohort of 662 patients diagnosed with RC at the same institution between Jan 2018 and Jun 2023, forming a distinct non-metastasis cohort. Inclusion criteria (a) and (b) mirrored those of the lung metastasis cohort, while criteria (c) were omitted in the non-metastasis cohort due to the absence of metastasis in these patients. The exclusion criteria remained consistent with those applied to the lung metastasis cohort.

Clinical information, including age, gender, pre-treatment histologic grade (24), and CEA levels, was extracted from medical records. It is important to note that CEA levels were determined through routine blood tests conducted within one week before treatment.

### MR imaging protocol

MRI was conducted using a 1.5-T MR system (Optimal 360, GE Healthcare, Waukesha, Wis) or a 3.0-T MR system (MR 750w, GE Medical System) with a phased-array body coil (eight-channel and sixteen-channel phased-array body coil). The standard procedure included axial oblique T2WI sequences and transverse DWI sequences. For the T2WI sequences, the in-plane pixel spacings ranged from 0.366 mm to 0.703 mm, with an average of 0.490 mm, and slice thicknesses ranged from 3.500 mm to 7.000 mm, with an average of 4.692 mm. For the DWI sequences, the in-plane pixel spacings ranged from 0.976 mm to 1.953 mm, with an average of 1.526 mm, and slice thicknesses ranged from 3.500 mm to 7.000 mm, with an average of 4.694 mm. Among the 819 patients in the lung metastasis cohort and non-metastasis cohort, images obtained from different scanners were randomly distributed in the training, validation, and independent test sets.

### Image pre-processing and segmentation

Radiologists with over 10 years of experience in MRI manually delineated the entire RC tumor using ITK-SNAP 3.9 on pre-treatment T2WI and pre-treatment DWI at *b*=1000 s/mm^2^. The resulting tumor masks were cropped into image patches as the inputs of 3D networks. To mitigate the impact of variability in acquisition and sequence parameters, image pre-processing was implemented before analysis. All MR images were used the Simple-ITK toolkit to correct the bias field artifacts (25). Gray-level normalization was applied to harmonize the gray values of MR images, compensating for intensity variations across different MRI scanners.

### Network details

**Figure 2:**
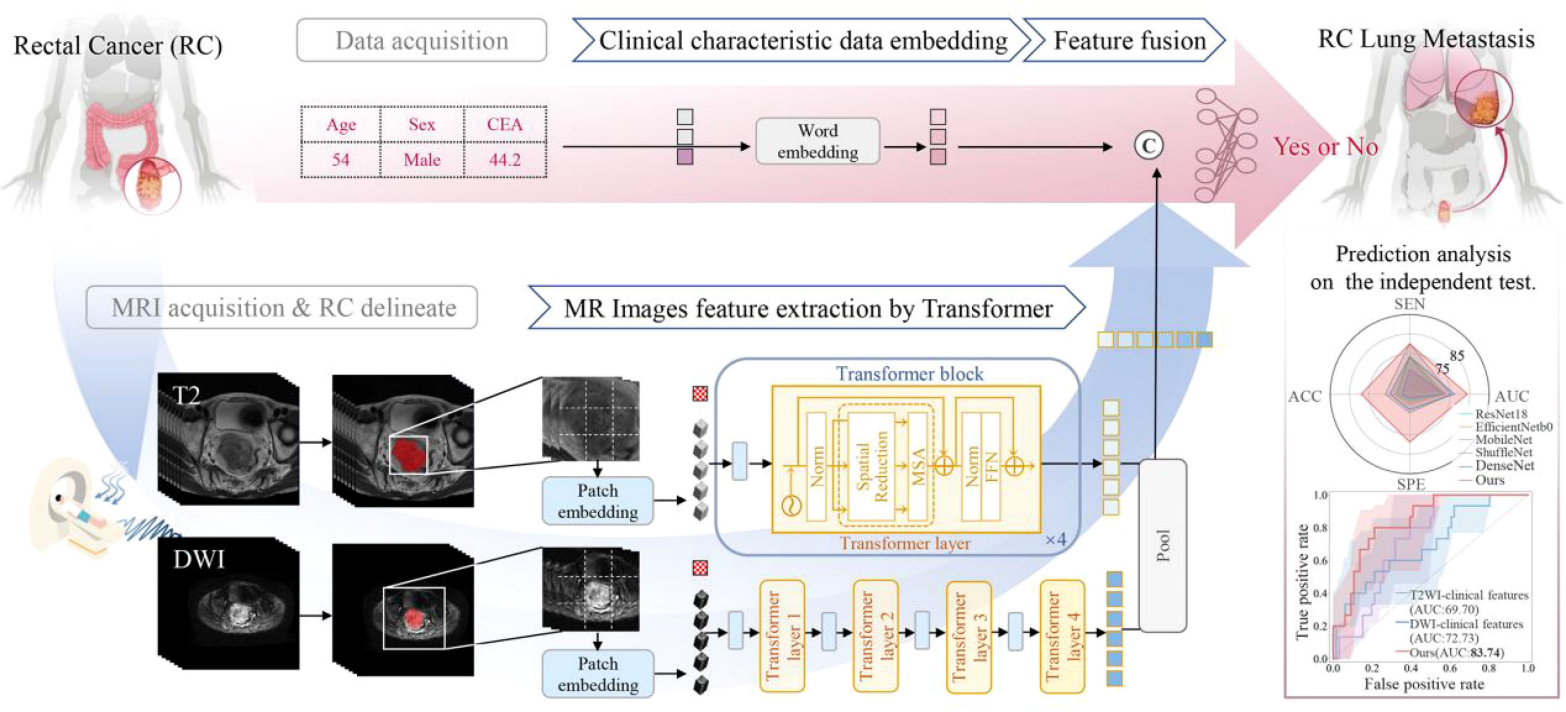
Overview of the proposed transformer-based deep learning model for rectal cancer with lung metastases.

The architecture of our proposed transformer-based DL model for predicting RCLM is illustrated in **Figure 2**. We used two pre-trained UniMiSS models, which were built upon the ViT framework, as our primary feature extraction network. T2WI and DWI scans underwent individual processing through each branch network, and the resulting features were fused through concatenation using a post-fusion approach. This fusion strategy engendered a comprehensive representation, combining complementary information from both T2WI and DWI. To distill spatial information, an adaptive max pooling operation was employed to reduce the feature length to 1000. These features were then concatenated with clinical information, including CEA level, age, and gender. The combined features were subsequently fed into a fully connected layers to effectively learn the imaging and clinical information for accurate predicting RCLM. This architecture enables the model to leverage both imaging and clinical data synergistically to enhance prediction performance.

### Data partitioning and network implementation

The performance of the models was evaluated on an independent test set, which was not included in the model training. The remaining data were randomly divided into training and validation sets for parameter tuning. Once optimal model parameters were determined using the training and validation sets, the models were evaluated on the independent test set. The models were implemented using PyTorch v1.7. Our models process 3D volumes, with a data batch size of 32 and input image dimensions of 128 (height) × 128 (width) × 16 (depth). In our preprocessing pipeline, we use manually annotated RC tumor masks to crop image patches from the 3D volume data to a specified size, while ensuring that the tumor region is fully retained. During the cropping process, the cropping area is dynamically adjusted based on the mask to ensure that the entire tumor is included within the cropped region. All MR images were linearly transformed to the range of [-1, 1] via gray-level normalization. Data augmentation techniques, including random rotation in the range of [-30, 30] and random scaling in the scale of [0.95, 1.05], were applied for model training to enhance generalization. For optimization, the Adam optimizer with an initial learning rate of 0.0002 was employed, and the models were trained for 100 epochs. Cross-entropy loss was used as the loss function for model training. To ensure a fair comparison, all compared models were trained using the same image size, learning rate, and number of iterations.

Among these, the model using only clinical characteristics employed the Logistic Regression model. The remaining five models of seven combinations of MRI sequences and clinical features used the pre-trained UniMiSS model (built on the ViT framework) to extract image features. Specifically, the “Images-only” model concatenated the DWI and T2WI images along the channel dimension before feeding them into the feature extraction network. When clinical features are included, a post-fusion method was applied to combine the image and clinical features. In addition, the two demographic characteristics (age and gender) and one CEA used in the comparison experiment were pre-treatment. Gender was labeled with 0 and 1, age was standardized with z-score, and CEA was scaled using minimum-maximum normalization. The latter two characteristics of information were subsequently transformed into one-dimensional representations via Word Embedding.

For a thorough evaluation, our method is compared with state-of-the-art deep learning models, including ResNet18, EfficientNetb0, MobileNet, ShuffleNet, and DenseNet. ResNet18 employs residual blocks with skip connections, enabling the network to learn residual functions. EfficientNetb0 uses a compound scaling method to balance depth, width, and resolution, optimizing performance and computational efficiency. MobileNet leverages depthwise separable convolutions to reduce complexity. ShuffleNet introduces a channel shuffle operation to enhance the efficiency of group convolutions while minimizing computational costs. DenseNet incorporates dense connections among all layers, fostering feature reuse and improving gradient flow, resulting in reduced parameters and enhanced training efficiency.

### Statistical analysis

All well-trained models were evaluated on both validation and independent test sets. The performance of the prediction model was assessed using various metrics, including the area under the receiver operating characteristic curve (AUC), accuracy (ACC), sensitivity (SEN), specificity (SPE) and the wilcoxon rank-sum test for statistical comparison. The optimal threshold for the AUC value was determined by maximizing the sum of the sensitivity and specificity values. The 95% CIs were obtained using bootstrapping to assess variability, and *p* < 0.05 considered indicative of statistically significant difference.

## Results

### Patient characteristics

Applying specific inclusion and exclusion criteria, our study enrolled a total of 819 patients, comprising 524 males and 295 females, with an average age of 57.25 ± 12.14 years (ranging from 17 to 86 years). The average CEA level among the patients was 12.58 ng/mL (ranging from 0.50 to 99.62 ng/mL). Among these patients, 657 were randomly assigned to the training set, 81 to the validation set, and 81 to the independent test set. Of the total patients, 157 patients (19.17%) were RC with lung metastasis, while 662 patients (80.83%) were RC without metastasis. In terms of tumor staging, there were 19 patients at the T1 stage (with 1 lung metastasis), 122 patients at the T2 stage (with 9 lung metastasis), 485 patients at T3 stage (with 82 lung metastasis), and 193 patients at T4 stage (with 65 lung metastasis). Regarding the N stage, there were 271 patients at the N0 stage (with 31 lung metastasis), 282 patients at the N1 stage (with 54 lung metastasis), and 266 patients at N2 stage (with 72 lung metastasis). The details of the demographic and clinical characteristics of patients were presented in **Table 1**.

**Table 1:** The details of the demographic and clinical characteristics of patients.

	Training Set	Validation Set	Independent test set
Variable	(n =657)	(n =81)	(n =81)
Sex			
F	227 (34.55%)	24 (32.88%)	36 (44.44%)
M	430 (65.45%)	49 (67.12%)	45 (55.56%)
Age			
Range	17-86	21-79	22-75
Average	57.33 ± 12.09	57.15 ± 11.99	56.68 ± 12.64
**Pretreatment CEA level (ng/mL)**	12.80 (0.50-99.62)	9.81 (0.95-83.79)	11.77 (0.50-64.12)
T stage			
T1	17 (2.59%)	2 (2.47%)	0 (0%)
T2	94 (14.31%)	14 (17.28%)	14 (17.28%)
T3	387 (58.90%)	50 (62.73%)	48 (59.26%)
T4	159 (24.20%)	15 (18.52%)	19 (23.46%)
N stage			
N0	222 (33.79%)	25 (30.86%)	24 (29.63%)
N1	220 (33.49%)	30 (37.04%)	32 (39.51%)
N2	215 (32.72%)	26 (32.10%)	25 (30.86%)
Metastasis situation			
Non-metastasis	530 (80.67%)	66 (81.48%)	66 (81.48%)
Lung metastasis	127 (19.33%)	15 (18.52%))	15 (18.52%)

Unless stated otherwise, data are numbers of patients, with percentages in parentheses. T stage = baseline clinical tumor stage; N stage = baseline clinical lymph node stage; CEA = carcinoembryonic antigen.

### Model performance on the validation and independent test sets

**Figure 3:**
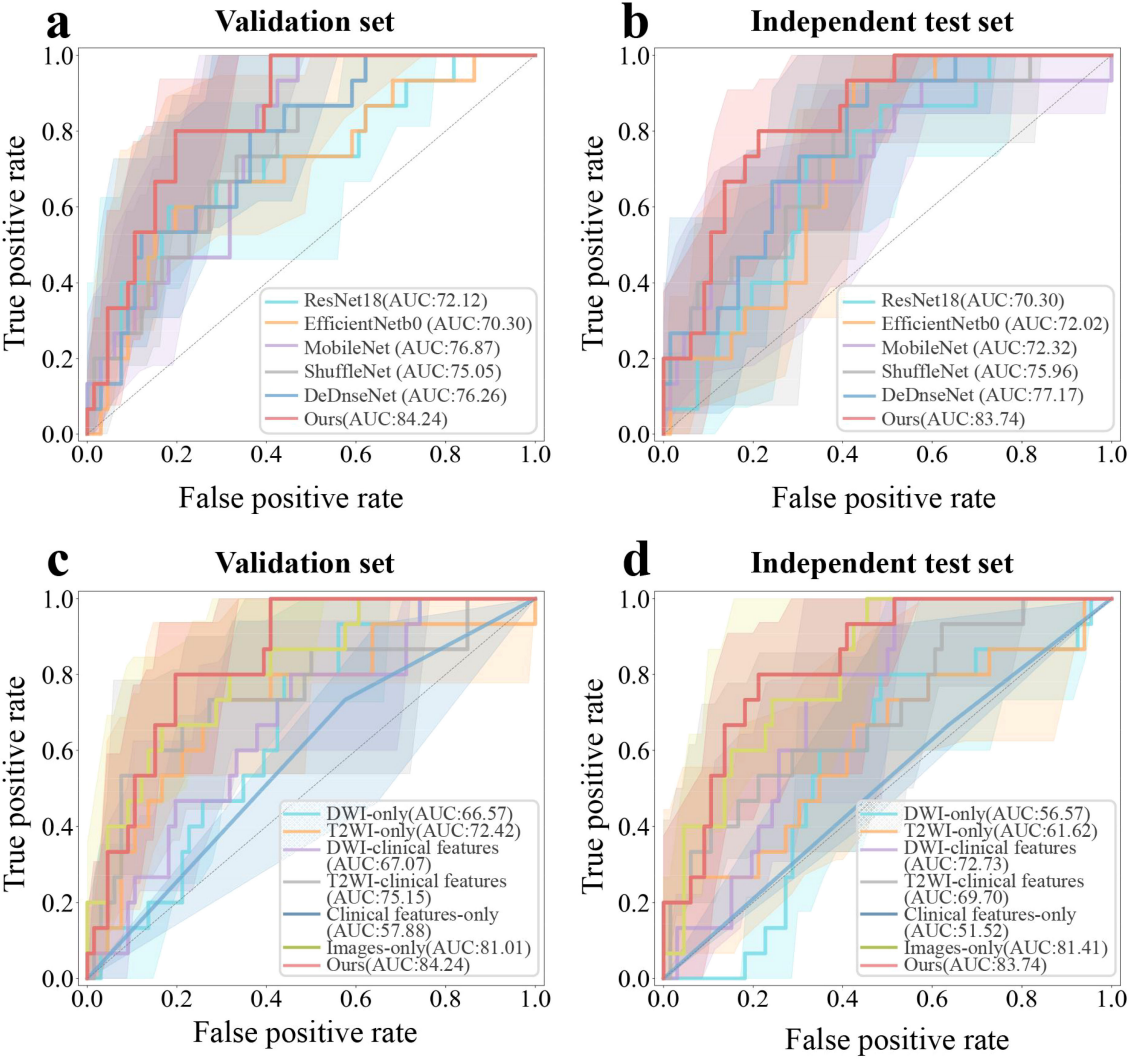
ROC curves of six distinct deep learning-based detection models, including ResNet18, EfficientNetb0, MobileNet, ShuffleNet, DenseNet and the proposed method, for rectal cancer with lung metastases (RCLM) detection on the validation set **(A)** and independent test set **(B)**; ROC curves of seven kinds of medical images and clinical features combination modes to detect RCLM on the validation set **(C)** and independent test set **(D)**.

We developed a transformer-based model for the prediction of RCLM. As shown in **Table 2**, the transformer-based model achieved an Area Under Curve (AUC) of 84.24% (95% CI, 73.87%-92.68%) on the validation set and 83.74% (95% CI, 72.60%-92.83%) on the independent test set. Our model outperformed the performance of ResNet18, EfficientNetb0, MobileNet, ShuffleNet, and DenseNet. **Figures 3A, B** illustrates the Receiver Operating Characteristic (ROC) curves for six deep learning models. These models yielded AUC values of 70.30% (95% CI, 56.18%-82.92%, *p* = 0.0196), 72.02% (95% CI, 58.73%-83.08%, *p* = 0.0186), 72.32% (95% CI, 56.36%-86.31%, *p* = 0.0158), 75.96% (95% CI, 61.84%-88.36%, *p* = 0.0495), 77.17% (95% CI, 64.26%-87.92%, *p* = 0.0412), and 83.74% (95% CI, 72.60%-92.83%) for ResNet18, EfficientNetb0, MobileNet, ShuffleNet, DenseNet and our method on independent test set, respectively.

**Table 2:** RCLM prediction performance obtained by the different DL models on both the validation and independent test set.

	Validation set				
Model	AUC (95% CI)	ACC	SEN	SPE	*P*_Value
ResNet18	72.12(54.65-86.67)	62.96	73.33	60.61	1.43E-02
EfficientNetb0	70.30(54.81-84.95)	59.26	66.67	57.58	1.96E-02
MobileNet	76.87(64.59-87.35)	65.43	73.33	63.64	3.30E-02
ShuffleNet	75.05(61.58-86.71)	62.96	73.33	60.61	1.05E-02
DenseNet	76.26(63.12-87.56)	65.43	73.33	63.64	4.12E-02
Ours	84.24(73.87-92.68)	80.25	80.00	80.30	–
	Independent test set				
ResNet18	70.30(56.18-82.92)	60.49	73.33	57.58	1.96E-02
EfficientNetb0	72.02(58.73-83.08)	61.73	73.33	59.09	1.86E-02
MobileNet	72.32(56.36-86.31)	58.02	66.67	56.06	1.58E-02
ShuffleNet	75.96(61.84-88.36)	65.43	73.33	63.64	4.95E-02
DenseNet	77.17(64.26-87.92)	64.20	73.33	62.12	4.12E-02
Ours	83.74(72.60-92.83)	79.01	80.00	78.79	–

P values were derived from the wilcoxon rank-sum test of comparing each metrics between different deep learning -based models and the proposed model.

**Table 3** presented the performance of our transformer-based model trained with seven combinations of MRI sequences and clinical features. These combinations include single image models (DWI-only or T2WI-only), models using a single image and clinical features (DWI-Clinical features or T2WI-Clinical features), a model using only clinical features (Clinical features-only), an image-only model with DWI and T2WI (Images-only), and a model using both DWI, T2WI, and clinical features (Images-Clinical features (Ours)). Incorporating two demographic characteristics (age and gender) and one clinicopathologic factors (CEA) into our model achieved the best performance. As outlined in **Table 3**, with increases of 2.86% in AUC, 23.07% in accuracy, and 30.00% in specificity compared to the original Image-only model on the independent test set, the combination of T2WI, DWI, and clinical features achieved the AUC of 83.74% (95% CI, 72.60%-92.83%) on the independent test set. **Figures 3C, D** illustrate the ROC curves for seven different combinations of image and clinical features. These combinations yielded AUC values of 56.57% (95% CI, 41.69%-71.22%, *p* = 0.0233), 61.62% (95% CI, 43.67%-78.57%, *p* = 0.0046), 72.73% (95% CI, 60.40%-83.75%, *p* = 0.0124), 69.70% (95% CI, 52.96%-84.55%, *p* < 0.001), 51.52% (95% CI, 37.73%-64.58%, *p* < 0.001), 81.41% (95% CI, 69.93%-90.72%, *p* = 0.0143), and 83.74% (95% CI, 72.60%-92.83%) for DWI-only, T2WI-only, DWI-clinical characteristics, DWI-clinical characteristics, clinical characteristics-only, images-only, and images-clinical characteristic on independent test set, respectively.

**Table 3:** Detection performance of seven combination models on the validation and independent test set.

	Validation set				
Model	AUC (95% CI)	ACC	SEN	SPE	*P*_Value
DWI-only	66.57(52.57-79.80)	58.02	60.00	57.58	2.33E-02
T2WI-only	72.42(55.56-87.12)	61.73	73.33	59.09	4.56E-03
DWI- Clinical features	67.07(52.17-80.07)	59.26	66.67	57.58	1.24E-02
T2WI-Clinical features	75.15(58.74-89.80)	56.79	73.33	53.03	2.08E-04
Clinical features-only	57.88(44.40-70.37)	48.15	73.33	42.42	2.13E-04
Images-only	81.01(68.78-91.34)	65.43	80.00	62.12	1.43E-02
Images-Clinical features (Ours)	84.24(73.87-92.68)	80.25	80.00	80.30	–
	Independent test set				
DWI-only	56.57(41.69-71.22)	55.56	66.67	53.03	9.02E-03
T2WI-only	61.62(43.67-78.57)	46.91	73.33	40.91	2.97E-04
DWI- Clinical features	72.73(60.40-83.75)	62.96	73.33	60.61	4.11E-02
T2WI-Clinical features	69.70(52.96-84.55)	54.32	66.67	51.52	4.68E-03
Clinical features-only	51.52(37.73-64.58)	41.98	66.67	36.36	2.47E-05
Images-only	81.41(69.93-90.72)	64.20	80.00	60.61	1.43E-02
Images-Clinical features (Ours)	83.74(72.60-92.83)	79.01	80.00	78.79	–

**Figure 4:**
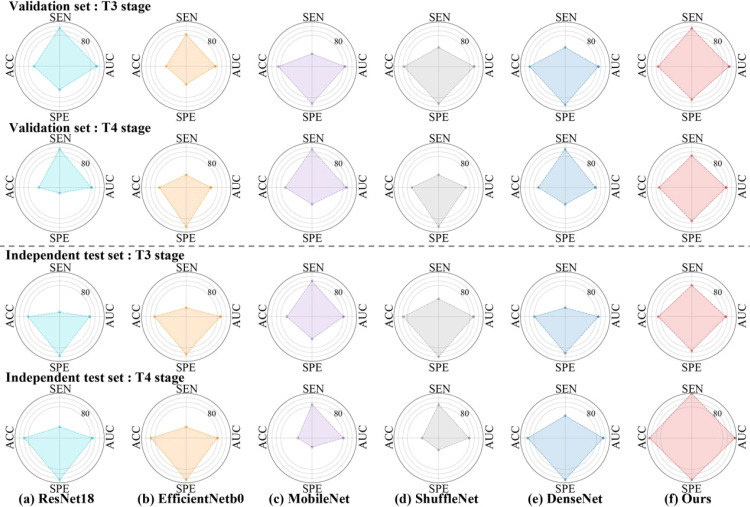
Radar plots that summarize the detection performance of the various models on the validation and independent test set. Characteristics of Patients Stratified by T3 and T4 stage. To test the efficacy of each algorithm, we calculated ACC = Accuracy, SEN = Sensitivity, SPE = Specificity, AUC= Area Under ROC Curve.

**Table 4** and **Figure 4** presented comprehensive results on both the validation set and the independent test set, focusing on different T and N stages. In **Table 4**, we provided a directly overview of various models’ performance at N stages. Furthermore, **Figure 4** visually illustrated the AUC, accuracy (ACC), sensitivity (SEN), and specificity (SPE) for stages T3 and T4 in different models. Our method outperformed the aforementioned state-of-the-art DL methods in terms of AUC in general. Specifically, we achieved an AUC of 76.32% (95% CI, 59.80%-89.55%) in the T3 stage, an AUC of 96.67% (95% CI, 87.14%-100%) in the T4 stage, an AUC of 85.00% (95% CI, 66.67%-100%) in the N0 stage, an AUC of 54.46% (95% CI, 29.89%-82.76%) in the N1 stage, and an AUC of 96.83% (95% CI, 88.67%-100%) in the N2 stage on the independent test set.

**Table 4:** Detection performance of the various models on the validation and independent test set.

	Validation set						Independent test set				
	Model	AUC (95% CI)	ACC	SEN	SPE	*P*_Value	AUC (95% CI)	ACC	SEN	SPE	*P*_Value
N stage											
N0	Resnet18	72.73 (25.00-100)	36.00	100	27.27	1.26E-02	81.25 (42.86-100)	54.17	75.00	50.00	1.96E-02
	EfficientNetb0	80.30 (45.00-100)	44.00	100	36.36	8.15E-03	73.75 (52.50-92.59)	58.33	100	50.00	1.14E-02
	MobileNet	71.21 (50.00-89.39)	40.00	100	31.82	2.09E-02	93.75 (80.95-100)	70.83	100	65.00	2.53E-02
	ShuffleNet	87.88 (72.62-100)	40.00	100	31.82	2.09E-02	87.50 (69.57-100)	66.67	100	60.00	3.39E-02
	DenseNet	72.73 (33.33-100)	40.00	100	31.82	1.14E-02	90.00 (75.00-100)	70.83	100	65.00	2.53E-02
	Ours	89.39 (62.50-100)	72.00	100	68.18	–	85.00 (66.67-100)	83.33	75.00	85.00	–
N1	Resnet18	71.25 (45.16-93.09)	47.37	75.00	40.00	1.26E-02	74.11 (53.33-92.86)	65.62	50.00	67.86	3.48E-02
	EfficientNetb0	71.67 (50.00-91.07)	47.37	75.00	40.00	2.01E-02	64.29 (36.61-87.10)	71.88	25.00	78.57	4.51E-03
	MobileNet	70.42 (51.67-87.50)	55.26	87.50	46.67	1.14E-02	68.75 (36.67-96.77)	71.88	50.00	75.00	1.26E-02
	ShuffleNet	74.17 (52.08-92.08)	52.63	75.00	46.67	3.48E-02	56.25 (25.00-87.10)	62.50	25.00	67.86	2.09E-02
	DenseNet	79.58 (60.61-94.79)	57.89	87.50	50.00	3.48E-02	72.32 (48.28-96.55)	68.75	50.00	71.43	2.09E-02
	Ours	82.50 (68.33-95.00)	76.32	87.50	73.33	–	54.46 (29.89-82.76)	50.00	75.00	46.43	–
N2	Resnet18	75.00 (42.22-100)	44.44	100	28.57	1.96E-02	61.11 (35.00-86.00)	52.00	85.71	38.89	1.96E-02
	EfficientNetb0	46.43 (0.00-86.67)	72.22	25.00	85.71	4.55E-02	65.08 (39.47-89.61)	52.00	85.71	38.89	3.48E-02
	MobileNet	91.07 (71.88-100)	50.00	100	35.71	3.39E-02	57.14 (25.74-87.00)	40.00	71.43	27.78	2.09E-02
	ShuffleNet	67.86 (35.29-100)	77.78	25.00	92.86	2.53E-02	80.95 (59.09-98.41)	64.00	100	50.00	1.43E-02
	DenseNet	69.64 (35.29-100)	38.89	100	21.43	4.68E-03	75.40 (48.00-96.10)	56.00	85.71	44.44	3.39E-02
	Ours	76.79 (39.29-100)	72.22	75.00	71.43	–	96.83 (88.67-100)	88.00	100	83.33	–

P values were derived from the wilcoxon rank-sum test of comparing each metrics between different deep learning -based models and the proposed model.

Characteristics of Patients Stratified by N stage.

### Performance of our model vs. experts

Three experts (Li X, Li B, Wan Y, with 32 years, 10 years and 4 years of experience, respectively) dedicated to imaging diagnosis based on MRI data in the independent test set. They consecutively and independently evaluated the MRI data from the independent test set. The diagnostic performance of subjective evaluation by three radiologists was presented in **Table 5**. The results of our model had been binarized for fair comparison. **Table 5** showed performance with AUCs of 56.52% (95% CI, 45.30%-69.65%, *p* < 0.001), 56.82% (95% CI,44.16%-70.89%, *p* < 0.001), 62.27% (95% CI, 47.88%-76.32%, *p* < 0.001) and 83.74% (95% CI,72.60%-92.83%) for three experts and our model. The diagnostic time for each case was 22.32s, 38.62s, 11.22s, and 0.67s for three experts and our model, respectively. As shown in **Table 5**, the experts’ results showed largely individual differences. In contrast, our model achieved the best performance in predicting RCLM.

**Table 5:** Detection performance by radiologists on the independent test set.

	AUC (95% CI)	ACC	SEN	SPE	*P*_Value	Time(s)
Reader 1	56.52 (45.30-69.65)	75.31	26.67	86.36	3.11E-04	22.32
Reader 2	56.82 (44.16-70.89)	71.60	33.33	80.30	1.62E-04	38.62
Reader 3	62.27 (47.88-76.32)	67.90	53.33	71.21	3.93E-04	11.22
Ours	83.74 (72.60-92.83)	79.01	80.00	78.79	–	0.67

P values were derived from the wilcoxon rank-sum test of comparing each metrics between radiologists and the proposed model.

## Discussion

This research aimed to investigate the potential of utilizing DL for RCLM detection based on pelvis MRI, with the goal of improving clinical decision-making, reducing radiation exposure, and mitigating treatment delays in patients without lung metastases. Our developed transformer-based model showed improved performance compared to five state-of-the-art DL models. Furthermore, the combination of T2WI and DWI MRI sequences with clinical features model achieved the best performance in both validation and testing sets.

Endorectal ultrasound has been used for the preoperative staging of early RC. However, its accuracy is highly dependent on operator experience and tumor size, which limits its clinical applicability (33). Positron emission tomography (PET)/CT provides valuable metabolic information but has relatively low spatial resolution and poor soft tissue contrast, which limits its sensitivity for detecting small lesions or early metastases (34). While PET/MRI can generate high-resolution anatomical and functional data with promising results for RC staging, it requires longer acquisition times and is more expensive (35). In our study, the combination of T2WI and DWI was chosen based on their complementary strengths: T2WI provides detailed structural information critical for local staging, while DWI enhances the detection of DM by offering functional insights. This combination was selected to maximize sensitivity and accuracy in detecting RC and its metastases, as demonstrated by the variations in sensitivity observed in **Table 3**.

As shown in **Table 1**, our dataset only contained only one RC patients with lung metastasis at T1 stage and 9 positive cases at T2 stage. Specifically, 9 patients with lung metastasis were randomly divided in the training set, resulting 2 cases in validation set and one case in testing set. Consequently, traditional metrics such as AUC, SEN, and SPE become inadequate for evaluating model performance when there are very few positive samples. Thus, we chose to focus on presenting results for RC patients at T3-T4 stages in **Figure 4**. Although our model demonstrated strong performance at T3-T4 stages, the limited sample size of T1-T2 stages posed challenges, resulting in modest information capture and average performance (26). Moreover, the locally advanced RC (T3-T4) is an important risk factor supporting lung metastasis diagnosis (3). Our transformer-based model yielded superior performance with an AUC of 76.32% (95% CI, 59.80%-89.55%) in the T3 stage and an AUC of 96.67% (95% CI, 87.14%-100%) in the T4 stage.

Existing state-of-the-art classification models for RCLM prediction suffer from field-of-view limitations due to the local receptive fields inherent in convolutions. Relying solely on local feature learning is insufficient for capturing the complex characteristics needed to predict lung metastases from pelvic MRI scans. In contrast, our Transformer-based models can capture global dependencies across image regions and integrate clinical features, resulting in more robust RCLM predictions. Our Transformer-based model offers several advantages over CNN-based methods. Firstly, our model consistently outperformed other state-of-the-art models, demonstrating its potential for higher diagnostic accuracy. Secondly, by integrating clinical features such as CEA levels, age, and gender, our model benefits from a holistic approach to prediction, which may help in capturing patient-specific factors that are important for accurate diagnosis.

Timely detection and intervention of lung metastasis play an important role in guiding clinicians in determining clinical decision-making, ultimately leading to enhanced R0 resection rates, reduced postoperative recurrence risks, and improved overall survival rates (27, 28). The method developed in this study provides the ability to predict RCLM based on pelvis MR images primary tumor. With its reliable information on lung metastasis detection, the model has the potential to alleviate the burden on clinicians and improve the efficiency of decision-making. Moreover, the model’s capability to perform these tasks based solely on MR images streamlines workflow and reduces dependence on other procedures, making it applicable in various clinical settings where MRI is routinely performed (29). The study’s findings demonstrated that the predictive performance of our model, leveraging image and clinical features of the primary tumor, surpassed the subjective evaluation by radiologists (**Table 5**).

Improving the accuracy of predicting distant metastasis in rectal cancer is crucial for informed clinical decision-making (10, 11). Previous research has predominantly focused on developing DL methods for predicting liver metastasis (21, 22, 30, 31) or lymph node metastasis (12, 15, 18) of RC. Specifically, there studies indicate that the imaging features of preoperative CT or MRI scans of RC have predictive values for the risk of distant metastasis. For instance, Lee et al. (22) proposed a CNN-based model incorporating clinical information and CT scans to predict liver metastasis in colorectal cancer that obtained an AUC of 74.70% (95% CI: 71.10%-78.30%). Numerous studies have shown that CEA is an essential indicator of recurrence and metastasis in patients with RC (14, 32). However, few studies have simultaneously combined MRI sequence of DWI and T2WI with CEA for predicting RCLM. In this study, we compared the performance of DL models using different combinations of medical images and clinical features in both the validation and independent test sets. Our findings suggest that utilizing the combination of images and clinical features achieves superior performance compared to those relying on a single feature in detecting lung metastasis in patients with RC.

Our study has several limitations. Firstly, the dataset was derived from a single center, which may affect the model’s generalizability. Future studies should prioritize incorporating multi-center data and additional prognosis-related data to strengthen external validation and ensure robust performance across diverse clinical settings. Secondly, the issue of sample imbalance, particularly for early-stage cases (T1 and T2), may have impacted the model’s predictive performance in these stages. Addressing this imbalance through targeted data collection or augmentation, such as synthetic data generation, will be crucial for future work. Thirdly, despite the superior performance on validation and independent test sets, the predictive scope is currently limited to lung metastasis. In our future studies, we will develop a DL model to predict various other types of metastasis. Fourthly, most cases in our dataset are locally advanced RC, and further research is needed to accurately identify distant metastasis in patients with early-stage RC. Additionally, we focused on integrating specific clinical features, such as CEA levels, age, and sex, due to their stronger association with predicting RCLM. However, we acknowledge that histologic grade may also provide valuable predictive information. We plan to investigate its potential inclusion in future studies to further enhance predictive accuracy. Lastly, RC lesion annotation by radiologists is time-consuming, and future studies should explore efficient automated segmentation networks for RC segmentation.

## Conclusions

In conclusion, we have developed a transformer-based DL model for predicting RCLM, relying on preoperative pelvis MRI scans and clinical features. Our model demonstrates superior performance compared to state-of-the-art DL models on both validation and independent test sets. It is anticipated that our model holds potential as a practical tool to reduce radiation exposure and mitigate treatment delays in patients without lung metastases, thereby supporting personalized treatment and follow-up plans.

## Data Availability

The original contributions presented in the study are included in the article/supplementary material. Further inquiries can be directed to the corresponding authors.
